# A Review on the Modeling of the Elastic Modulus and Yield Stress of Polymers and Polymer Nanocomposites: Effect of Temperature, Loading Rate and Porosity

**DOI:** 10.3390/polym14030360

**Published:** 2022-01-18

**Authors:** Reema H. Alasfar, Said Ahzi, Nicolas Barth, Viktor Kochkodan, Marwan Khraisheh, Muammer Koç

**Affiliations:** 1 Division of Sustainable Development, College of Science and Engineering, Hamad bin Khalifa University, Doha P.O. Box 34110, Qatar; mkoc@hbku.edu.qa; 2 Mechanical Engineering Program, Texas A&M University at Qatar, Doha P.O. Box 34110, Qatar; ahzisaid@gmail.com (S.A.); marwan.khraisheh@qatar.tamu.edu (M.K.); 3 Qatar Environment and Energy Research Institute, Hamad bin Khalifa University, Doha P.O. Box 34110, Qatar; nbarth@hbku.edu.qa (N.B.); vkochkodan@hbku.edu.qa (V.K.)

**Keywords:** modeling, polymers, polymer nanocomposites, elastic modulus, yield stress, porosity effect, strain rate effect, temperature effects

## Abstract

Porous polymer-based nanocomposites have been used for various applications due to their advantages, including multi-functionalities, easy and known manufacturability, and low cost. Understanding of their mechanical properties has become essential to expand the nanocomposites’ applications and efficiency, including service-life, resistance to different loads, and reliability. In this review paper, the focus is on the modeling of the mechanical properties of porous polymer-based nanocomposites, including the effects of loading rates, operational temperatures, and the material’s porosity. First, modeling of the elastic modulus and yield stress for glassy polymers and polymer reinforced by nanofillers are addressed. Then, modeling of porosity effects on these properties for polymers are reviewed, especially via the use of the well-known power-law approach linking porosity to elastic modulus and/or stress. Studies related to extending the mechanical modeling to account for porosity effects on the elastic modulus and yield stress of polymers and polymer-nanocomposites are discussed. Finally, a brief review of the implementation of this modeling into 3D computational methods to predict the large elastic-viscoplastic deformation response of glassy polymers is presented. In addition to the modeling part, the experimental techniques to measure the elastic modulus and the yield stress are discussed, and applications of polymers and polymer composites as membranes for water treatment and scaffolds for bone tissue engineering are addressed. Some modeling results and validation from different studies are presented as well.

## 1. Introduction

Polymers have been widely used and considered for a variety of applications due to their advantages such as multi-functionalities, easy and known manufacturability, and low cost. In order to broaden their applications, increase their functionalities, reliability, durability, and cost-effectiveness, polymers are reinforced with fillers to improve their stiffness and strength. Fillers in the nanoscale form are commonly acknowledged to enhance mechanical, thermal, electrical properties, etc. Nanofillers have at least one length scale in the order of nanometers [1]. The morphologies of nanofillers vary from isotropic to highly anisotropic, and uniform dispersion of isotropic and anisotropic nanofillers results in achieving a very large interfacial area/volume between the nanofillers and the polymer matrix [2]. This large interfacial area makes a polymer nanocomposite have superior properties compared to the polymer itself.

Nanofillers can enhance the mechanical properties, including strength, stiffness, and toughness, and they can also improve the thermal and electrical conductivities of the polymers [3,4]. They are also able to add functionality for a particular application [4].

**Figure 1:**
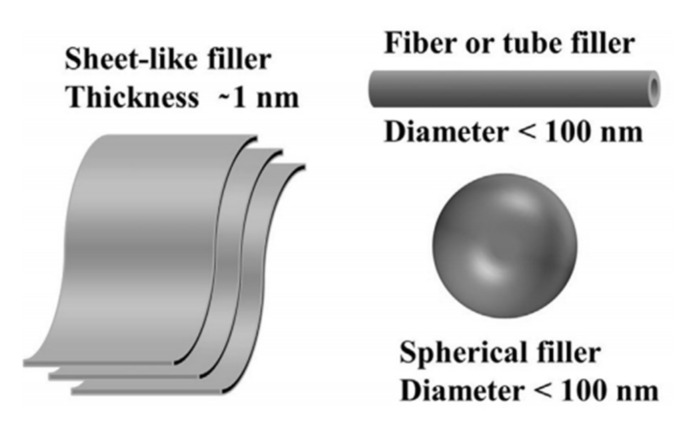
The classification of nanofillers. Reprinted with permission from [2].

Nanofillers can be classified, see Figure 1, according to the number of dimensions that are not in the range of nanoscale (<100 nm), as follows [1,2]:

1. Zero-dimensional nanofillers: all dimensions are at the nanoscale (<100 nm). 0-D nanofillers are also known as nanoparticles;
2. One-dimensional nanofillers: one dimension only is not at the nanoscale. Such materials include nanotubes, nanofibers, nanowires, and nanorods, e.g., carbon and halloysite nanotubes;
3. Two-dimensional nanofillers: exactly two dimensions are not at the nanoscale. These include nanofilms and nanoplates/sheets, e.g., graphene sheets and layered silicate.

**Figure 2:**
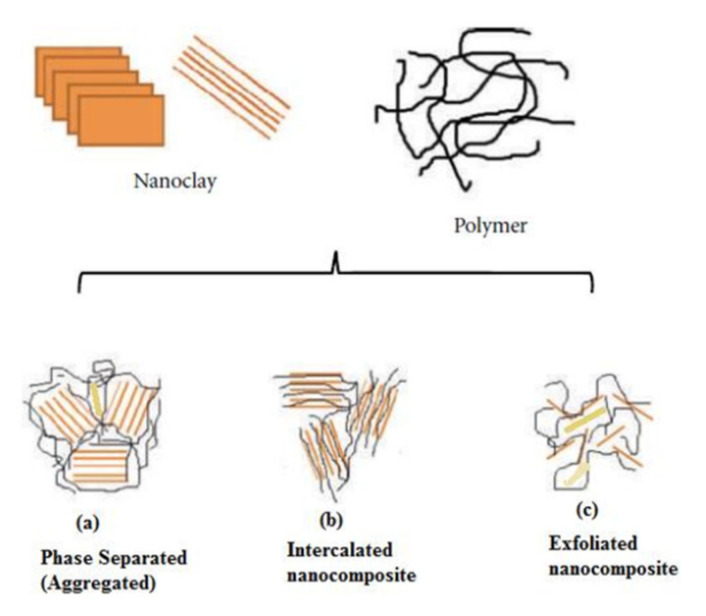
The dispersion of layered nanoclay. Reprinted with permission from [5].

As defined previously, the two-dimensional nanofillers are the sheets-like or layered nanofillers. When integrated into the polymer matrix, the microstructure of layered polymer nanocomposites can be further categorized into: (1) phase-separated (aggregated), (2) intercalated, and (3) exfoliated, as shown in Figure 2 [5].

The focus of this paper is to present a comprehensive and comparative review and analysis of the mechanical properties of polymer nanocomposites, with both aspects of characterization and modeling approaches. The mechanical properties of polymer nanocomposites are affected by the dispersion of a nanomaterial within a polymer matrix. An exfoliated nanocomposite has a much higher strength than an intercalated nanocomposite [2,5]. This is mainly because of the higher degree of contact between the exfoliated nanomaterial and the polymer [3]. Cho and Paul [6] have prepared Nylon 6/organoclay nanocomposites and by using transmission electron microscopy (TEM), a well-dispersed organoclay in the matrix was observed. Their study indicated that the modulus and yield strength of the well-exfoliated nanocomposites have increased with increasing the organoclay content [6]. Liu, Qi, and Zhu [7] have prepared nylon 6/clay exfoliated nanocomposites by a melt-intercalation process and showed that the yield strength, flexural strength, flexural modulus, and tensile modulus have all significantly increased with the addition of a small weight percentage of nanoclay (4.2 wt.%). They have reported that this is because of the strong interaction between the nanoclay interface and the nylon 6 matrix, which was indicated by the TEM and X-ray diffraction (XRD) [7]. Ma, Zhang, and Qi [8] have prepared intercalated clay (MMT)/elastomeric polyurethane (MMT/PU) nanocomposite and studied the effect of increasing the clay content on the tensile strength. They have illustrated that the strength of the nanocomposite has increased as the clay content increased from 0 to 8 wt.% [8]. However, the strength starts to decline for a clay content higher than 8 wt.%, which is due to the aggregation of clay as illustrated by wide-angle X-ray diffraction [8]. Hassanzadeh-Aghdam and Mahmoodi [9] have shown that incorporation of SiO_2_ nanoparticles into shape memory polymer (SMP) results in enhancement of the elastic modulus of the nanocomposite. They concluded that it is essential to account for the interphase region in the micromechanical modeling to obtain an accurate prediction of the nanocomposite elastic modulus [9]. Furthermore, Xie et al. [10] have determined the degree of clay dispersion based on TEM and optical microscopy data. They have shown that an increase in the storage modulus is achieved by enhanced dispersion of the nanoclay in the polypropylene (PP) nanocomposite.

The effect of nanofiller agglomeration on the effective properties of nanocomposites is significant. Haghgoo et al. [11] have predicted the electrical conductivity for carbon fiber-carbon nanotubes (CNT)-polymer hybrid composite using a two-step analytical method. They have shown that at a higher CNT volume fraction, the agglomeration effect is higher due to the formation of more CNT clusters. Higher electrical conductivity is achieved in the agglomerated state and can be explained by the increase in CNT-CNT contacts in the clusters [11]. In another study by Haghgoo et al. [12], they have concluded the importance of the effect of agglomerate size to tunneling distance ratio on the electrical conductivity. A more recent study by Haghgoo et al. [13] also revealed that the electrical conductivity increases with the increase in agglomeration size. In terms of thermal conductivity, Hassanzadeh-Aghdam and Ansari [14] have shown that CNT agglomeration lowers the thermal conductivity of SMP nanocomposites. Hence, it is crucial to have a uniform dispersion of the CNT to enhance the thermal properties.

In addition, Hassanzadeh-Aghdam [15] has illustrated the impact of agglomeration on the creep modulus of graphene nanoplatelet (GNP)-reinforced epoxy nanocomposite. A homogenization approach based on Mori-Tanaka micromechanical model was used. To have acceptable predictions, it is important to account for the GNP agglomeration in the micromechanical analysis as the graphene content increases (i.e., the formation of agglomeration increases) [15]. In another study done to predict the creep behavior of CNT polymer nanocomposite (PNC), it was concluded that the dispersion type, directional behavior of CNTs, and the interphase region are all factors that must be considered to achieve a more realistic prediction of the CNT-PNC creep response [16]. Shi et al. [17] have studied the effect of agglomeration of CNTs on the effective stiffness of the composite using Eshelby’s inclusion model. They illustrated that the agglomeration of CNTS resulted in reducing the effective elastic modulus. Ji et al. [18] have studied the factors that reduce the elastic modulus of graphene sheet-reinforced polymer nanocomposites, which are agglomeration, restacking, and scrolling of graphene sheets. Furthermore, Kundalwal and Ray [19] have theoretically investigated the effect of the interphase between the CNTs and the matrix on the effective properties of the composite. They concluded that the effect of the interphase on the longitudinal effective elastic properties was negligible. However, the study by Snipes et al. [20] showed the importance of considering the interface region, between the matrix and fillers phases, on the polymer nanocomposites’ stiffness.

To enhance the mechanical properties of a polymer nanocomposite, it must have a good dispersion of nanofillers in the polymer matrix [2,21,22]. In addition, the interfacial interaction between the polymer matrix and the nanofillers plays a significant role in the mechanical properties of the polymer nanocomposites [2]. A strong interfacial interaction usually results in better mechanical properties of the nanocomposites [2,21]. This is not only shown through experiments but also specifically accounted for with theoretical modeling, which will be discussed more in detail in this paper.

## 2. Modeling the Mechanical Behavior of Polymers and Polymer Nanocomposites

**Figure 3:**
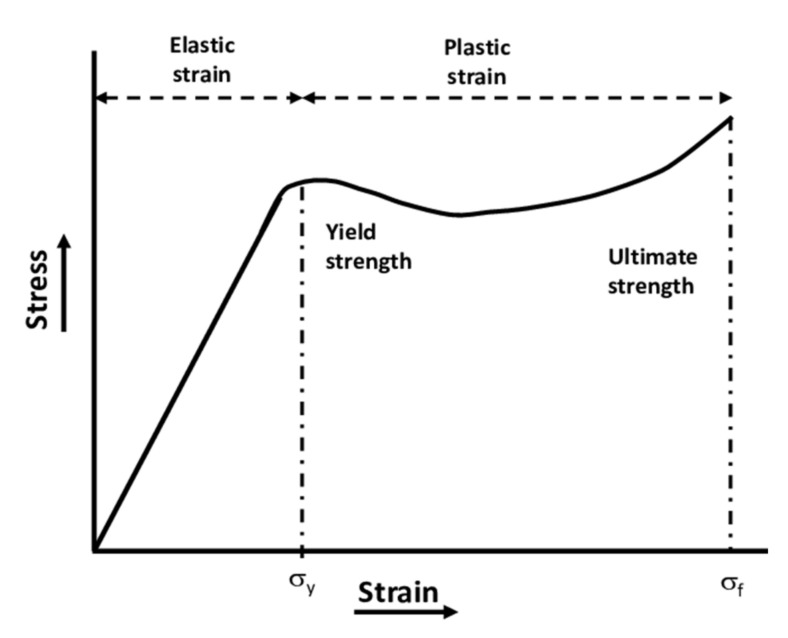
Typical stress-strain curve of an amorphous polymer. Reprinted with permission from [23].

It is essential to define the mechanical properties that describe the behavior of polymers before discussing any mechanical modeling. Figure 3 illustrates a schematic of the typical stress-strain curve for a polymer below the glass transition temperature [23]. The curve starts by a linear elastic region where the slope is the elastic modulus. The modulus of elasticity, or Young’s modulus, is defined as the ratio of stress (σ) to strain (ε) in the linear elastic region (i.e., E=Δσ/Δε). It is a measure of the stiffness of the material. As seen in Figure 3, the yield strength ($\sigma_{y}$), or the onset of yielding, is the stress that corresponds to the end of the elastic region. Within the elastic region, the deformation is reversible, which means the material completely recovers its original dimension [24]. Hence, in this region, the linear relationship that exists between the stress and strain is Hooke’s law where: σ=Eε [25].

However, in the plastic region, the deformation is not recoverable; it is permanent [24]. The behavior, as shown in Figure 3, typically starts with strain softening followed by strain hardening where neck propagation takes place [26,27]. The ultimate strength, or the tensile strength, shown in Figure 3, corresponds to the maximum stress before the polymer fractures. The ultimate elongation or elongation to break is a measure, in percentage, of the change in length in the material before the fracture. Compared to polymers, ceramics have a very low elongation to break, and metals have a moderate elongation to break. The area under the stress-strain curve gives the toughness of the material [24].

**Figure 4:**
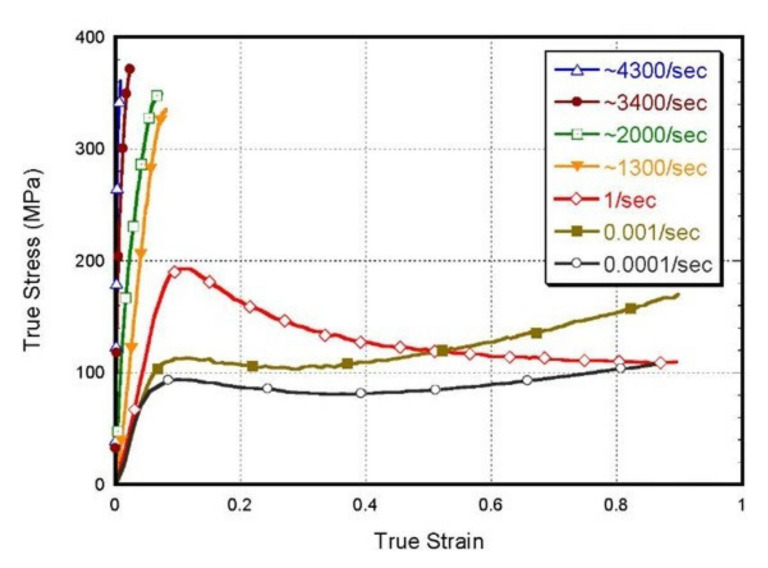
Influence of strain rate on the stress-strain behavior of poly(methyl methacrylate) (PMMA). Reprinted with permission from [30].

In several aspects, polymeric materials are mechanically different from metallic and ceramic materials. As an example, the maximum tensile strength of a metal alloy can reach up to 4100 MPa, while the maximum tensile strength of polymers is around 100 MPa [26]. Although metals are sensitive to high strain rate and temperature, the mechanical properties of polymers are highly sensitive to changes in strain rate and temperature [28]. William and Rethwisch [26] have discussed the influence of changes in temperature on the stress-strain behavior of poly(methyl methacrylate) (PMMA). They illustrated that the rise of temperature (from 4 °C to 60 °C) leads to a decrease in elastic modulus and yield strength, and an increase in ductility. However, the increase in the strain rate has opposite effects on the stress-strain behavior of polymers to the increase in temperature [29]. As shown in Figure 4, increasing the strain rate leads to an increase in elastic modulus and yield strength of PMMA and reduction in ductility [30].

### 2.1. General Modeling and Simulation Methods

**Figure 5:**
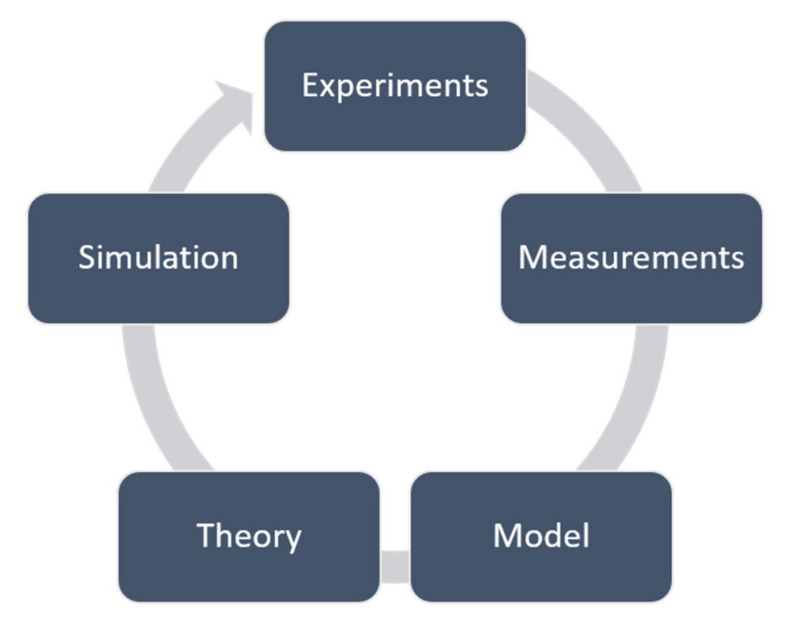
A schematic showing the interdependence between modeling and experimental methods (adapted from Ref. [32]).

Different modeling tools exist that predict the mechanical properties of polymeric materials and polymeric nanocomposite materials [31]. In general, the role of modeling can be schematically viewed in Figure 5. Laboratory experiments are critical for the validation of the model, and measurements are necessary to obtain certain parameters needed for the model [32,33]. A model is used to develop the essential theory, which is used to compare experimental results to the predicted behavior through simulation [33].

According to Valavala and Odegard [33], computational modeling methods used for the prediction of mechanical properties of materials can be divided into computational chemistry and computational mechanics methods. Both analytical and numerical methods are continuum-based methods [34]. Numerical methods include both finite and boundary element methods (FEM and BEM) [33]. All the computational modeling methods described in this paper are considered analytical micromechanics methods.

### 2.2. Elastic Behavior of Polymers

Temperature and strain rate significantly affect the mechanical properties of a polymer [21,22,35,36]. The elastic behavior of polymers changes significantly as the temperature and strain rate change [37]. As a result, it is necessary to have a model that considers the effect of temperature and strain rate on the modulus of elasticity. The modulus of elasticity or Young’s modulus, is an essential property in polymers. It is the ratio of stress to the elastic strain, and it depends on temperature and strain rate. Richeton et al. [35] have developed a model for the stiffness modulus, which takes into account the effect of temperature and strain rate/frequency. The basis of their work is the statistical model for modulus dependency on temperature, which was developed by Mahieux and Reifsnider [38,39]. The Mahieux and Reifsnider model is the only model which is valid from fully glassy to fully rubbery polymer materials [35]. Their approach is based on the effect of temperature on bonding in polymers, particularly secondary bonds [38].

**Figure 6:**
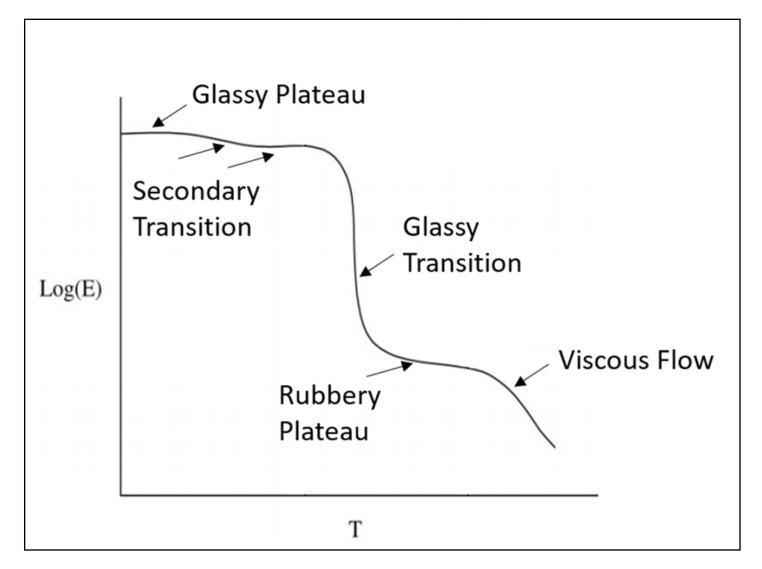
Log (modulus) vs. temperature for an amorphous polymer. Reprinted with permission from [38].

In fact, in polymers, from the glassy to the rubbery regions, the modulus of elasticity drops from gigapascal to megapascal [39]. Hence, it is important to examine how Young’s modulus for a polymer changes with temperature. Figure 6 shows the log of modulus (*E*) versus temperature (T) for a typical linear amorphous polymer [38]. As illustrated in Figure 6, there are five regions in the curve, which are [40]:

(1) The glassy region where the modulus is high (in GPa);
(2) The glass transition region where the modulus sharply goes down;
(3) The rubbery region where the modulus is low (in MPa);
(4) The viscous region where a polymer begins to flow;
(5) Decomposition region where the chemical breakdown begins.

**Figure 7:**
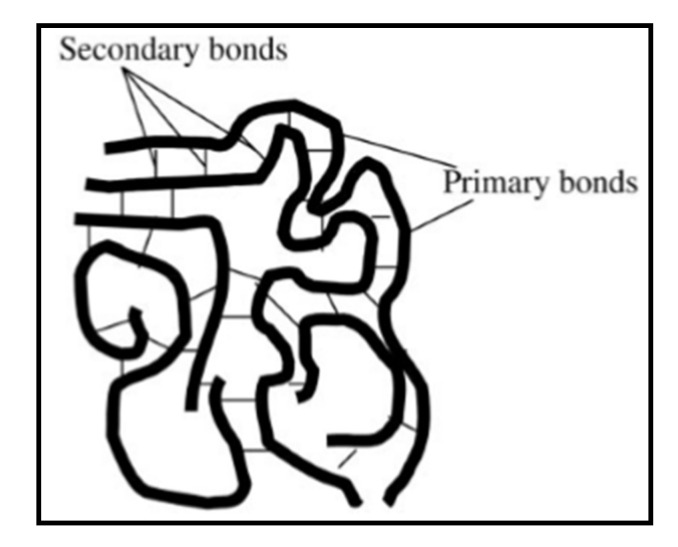
Primary and secondary bonds in polymer. Reprinted with permission from [38].

Before discussing these regions, note that there are primary and secondary bonds in the polymer. The primary bonds in the polymer do not break down nor dissociate as the polymer goes from the glassy to viscous flow region [39]. The primary bonds in polymer macromolecules are strong covalent bonds with the energy of dissociation ranging from 50 to 200 kcal/mol [38]. On the other hand, the secondary bonds in polymers are weaker: such as Van der Waals interactions (0.5 to 2 kcal/mol), dipole interactions (1.5 to 3 kcal/mol), hydrogen bonds (3 to 7 kcal/mol), and ionic bonds (10 to 20 kcal/mol) [38]. The strength of the secondary bonds changes as the polymer goes from the glassy to the viscous flow region. Figure 7 shows the primary and secondary bonds in polymers [38].

(1) The glass region and secondary relaxation

The glass temperature ($T_{g}$) can be defined as the temperature where the secondary bonds begin to dissociate. When the polymer is loaded at a temperature less than $T_{g}$, the secondary bonds stretch [40]. This elastic deformation is recovered after the load is removed.

In the glassy region, the modulus of elasticity is the highest, as shown in Figure 6. If the thermal energy is high, this may lead the side groups to rotate in the polymer macromolecules [38]. In this case, the secondary relaxation is viewed by a decrease in the modulus, as shown in Figure 6. This kind of rearrangement requires a low amount of activation energy, and therefore, these relaxations can happen at a temperature below $T_{g}$ [38].

(2) The glass transition region

As illustrated in Figure 6, the glass transition leads to a significant drop in the modulus of elasticity. Loading the polymer at $T_{g}$ or above $T_{g}$ results in the movement of the macromolecules [41]. The molecules slide in a way known as “reptation” which is usually referred to as a “snake-like way” as described by Ashby [40]. As a result of molecules’ “reptation”, the secondary bonds begin to break [38].

However, it is important to mention that the movement of polymer macromolecules is still limited as a result of cross-linking/entanglements of the polymer chains, or presence of crystallites, and fillers within the polymer matrix, etc. [40,41]. Additionally, in this region, there is the elastic part, which will restore some of the original shapes of the polymer after unloading. However, the work of the elastic part takes time since it will pull against the movement (sliding) of molecules; hence, the polymer has “leathery properties” [40].

(3) The rubbery region

After the glass transition, polymers with a long chain (average degree of polymerization > 10^4^) go through the rubbery region. The rubbery state of polymers is a result of the entanglement of the polymer macromolecule [40].

Above $T_{g}$, the entanglements act as “a shape-memory”. When the polymer is loaded, the entanglements straighten out and when unloaded, the straightened polymeric chains contract and use the entanglements to pull the polymer to its original shape [40]. The modulus of elasticity in the rubbery region is low compared to the glassy region.

(4) Viscous flow region

At the viscous region, the temperature is high (>1.4 $T_{g}$) and the modulus of elasticity is low [41]. At this stage, the secondary bonds in the polymer macromolecules had fully dissociated and the points of entanglements had slithered [38]. Hence, linear polymers are viscous liquids at this stage [38]. However, cross-linked polymers will not melt, but they will decompose at a very high temperature. In addition, the class of thermoplastic polymers are actually shaped at the viscous flow region [40].

(5) Decomposition region

Decomposition of polymers into monomer units (e.g., poly(methyl methacrylate) (PMMA)) or into several products (e.g., polyethylene (PE)) occurs at a very high temperature. As a result, it is vital to ensure that thermoplastic polymers are not overheated when they are shaped to avoid the decomposition of the polymer [38].

Figure 6 illustrates the changes in modulus in the five different regions for a typical linear amorphous polymer. Crystallization in polymers increases the Young’s modulus since the molecules in crystalline polymers are “more densely packed” compared to amorphous polymers [40,41]. Furthermore, cross-linked polymers have a higher Young’s modulus in the rubbery region. However, cross-linked polymers do not melt, unlike crystalline polymers, which melt at a high temperature [40].

### 2.3. Mahieux and Reifsnider Model for the Elastic Modulus

The Mahieux and Reifsnider [38] model is valid from the glassy to the rubbery region and it includes the three transition temperatures (beta transition ($T_{\beta }$), glass transition ($T_{g}$), and flow ($T_{f}$)) as well as the three instantaneous stiffnesses at the beginning of each region. The authors used the Weibull moduli ($m_{i}$) to represent the “statistics of the bond breakage”. The modulus as a function of temperature, $E\left(T\right)$, is:

(1) $$\begin{array}{c} E\left(T\right)\;=\;\left(E_{1}-E_{2}\right)\cdot \exp\left(-{\left(\frac{T}{T_{\beta }}\right)}^{m_{1}}\right)\;+\;\left(E_{2}-E_{3}\right)\cdot \exp\left(-{\left(\frac{T}{T_{g}}\right)}^{m_{2}}\right)+\\ E_{3}\cdot \exp\left(-{\left(\frac{T}{T_{f}}\right)}^{m_{3}}\right). \end{array}$$

Here, each subscript (1,2,3) refers to a particular transition (1 for beta, 2 for glass transition, 3 for flow transition). The instantaneous modulus $E_{i}$ is the modulus of each region just before the transition. The Weibull parameter $m_{1}$ affects the slope of the secondary relaxation, $m_{2}$ affects the slope of the glass transition region, and the last Weibull parameter $m_{3}$ affects the slope of the flow region [38]. These Weibull parameters are then important to define the behavior of the material of interest.

In another paper by Mahieux and Reifsnider [39], they applied the model into four polymers: polyether ether ketone (PEEK), PMMA, polyphenylene sulfide (PPS) and the composite AS4/PPS. The model was found to correctly represent all of the four polymers’ responses. However, as clearly shown in the above model, the effect of frequency/strain rate on modulus is not considered. Therefore, Richeton et al. [35] have modified the above model to account for the influence of temperature and frequency/strain rate as described in the next section.

### 2.4. Richeton Model for the Elastic Modulus

Richeton et al. [35] relied on the model of Mahieux and Reifsnider and included the frequency or strain rate as follows on the parameters under focus:

The storage modulus is expressed as [35]:

(2) $$\begin{array}{c} E\left(T,f\right)\;=\;\left(E_{1}\left(f\right)\;-\;E_{2}\left(f\right)\right)\cdot \exp\left(-{\left(\frac{T}{T_{\beta }\left(f\right)}\right)}^{m_{1}}\right)\;+\;\left(E_{2}\left(f\right)\;-\;E_{3}\left(f\right)\right)\cdot \\ \exp\left(-{\left(\frac{T}{T_{g}\left(f\right)}\right)}^{m_{2}}\right)\;+\;E_{3}\left(f\right)\cdot \exp\left(-{\left(\frac{T}{T_{f}\left(f\right)}\right)}^{m_{3}}\right), \end{array}$$

where all terms were defined for Equation (1). Similarly, for Young’s modulus, the frequency (f) is replaced by the strain rate ($\dot{\varepsilon }$) in Equation (2) [35]:

(3) $$\begin{array}{c} E\left(T,\dot{\varepsilon }\right)\;=\;\left(E_{1}\left(\dot{\varepsilon }\right)\;-\;E_{2}\left(\dot{\varepsilon }\right)\right)\cdot \exp\left(-{\left(\frac{T}{T_{\beta }\left(\dot{\varepsilon }\right)}\right)}^{m_{1}}\right)\;+\;\left(E_{2}\left(\dot{\varepsilon }\right)\;-\;E_{3}\left(\dot{\varepsilon }\right)\right)\cdot \\ \exp\left(-{\left(\frac{T}{T_{g}\left(\dot{\varepsilon }\right)}\right)}^{m_{2}}\right)\;+\;E_{3}\left(\dot{\varepsilon }\right)\cdot \exp\left(-{\left(\frac{T}{T_{f}\left(\dot{\varepsilon }\right)}\right)}^{m_{3}}\right). \end{array}$$

In these expressions, the instantaneous moduli and transition temperatures have the following dependence on the frequency or strain rate [35]:

(4) $$E_{i}=E_{i}^{ref}\cdot \left[1+s\cdot \log_{10}\left(\frac{f}{f^{ref}}\right)\right],$$

where $i=\left\{1,2,3\right\},$ respectively, for the three aforementioned transitions $\left\{\beta ,\mathrm{glass},\mathrm{flow}\right\}$, “*ref*” in the superscript indicates a reference value, and s is the sensitivity constant of the modulus to frequency for a specified polymer.

(5) $$\frac{1}{T_{\beta }}=\frac{1}{T_{\beta }^{ref}}+\frac{k}{\Delta H_{\beta }}\cdot \ln(\frac{f^{ref}}{f})\;,$$

(6) $$T_{g}=T_{g}^{ref}+\frac{-c_{2}^{ref}\cdot \log\left(f^{ref}/f\right)}{c_{1}^{ref}+\log\left(f^{ref}/f\right)},$$

(7) $$T_{f}=T_{f}^{ref}\cdot \left(1+0.01\log\left(\frac{f}{f^{ref}}\right)\right)\;,$$

where k is the Boltzmann constant, $\Delta H_{\beta }$ is the activation energy, and $c_{1}^{ref}$ and $c_{2}^{ref}$ are the Williams-Landel-Ferry (WLF) parameters [35,42].

For both storage and Young’s moduli, the following equivalence can be applied [35]:

(8) $$\frac{f^{ref}}{f}=\frac{{\dot{\varepsilon }}^{ref}}{\dot{\varepsilon }}.$$

Richeton et al. [35] have validated the model with their experimental work by applying it to two polymers: PMMA and PC. Their results of the dynamic mechanical analysis (DMA) showed a good agreement with the model for the storage modulus for both polymers under a wide range of frequency. In addition, the initial Young’s modulus obtained from the uniaxial compression test was in agreement with the model results.

In a recent paper, Çolak and Çakir [43] have determined the material parameters for the storage modulus in Equation (2) via a genetic algorithm (GA) optimization method. They have proven that the procedure is successful for two thermoplastic polymers: one amorphous (plasticized PVC) and the other semi-crystalline (PP). This was done by comparing the modeling results to experimental DMA data. In another paper by these authors [44], the material parameters for epoxy resin were successfully determined using the GA optimization method and they have shown the agreement between the predicted storage modulus and experimental data.

## 3. Elastic Behavior of Polymer Nanocomposites

The Richeton et al. [35] model considered the effect of temperature and frequency/strain rate, and it is a model that is valid from fully glassy to fully rubbery region. Hence, it is a significant statistical model that can be used to determine the elastic properties of polymers. However, it is of great importance to have a model for polymer nanocomposites since they are needed for numerous applications, such as in the field of water polymeric membranes.

### 3.1. Halpin-Tsai Model

The Halpin-Tsai model is a semi-empirical model that has been developed by Halpin and Kardos [45] to estimate the elastic modulus of composite materials. The Halpin-Tasi Equation can be written as the following [46]:

(9) $$\frac{E}{E_{m}}=\frac{1+\xi \eta \varphi_{f}}{1-\eta \varphi_{f}}\;\;.$$

Here, E is the longitudinal modulus ($E_{L}$) or the transverse modulus ($E_{T}$), $E_{m}$ is the elastic modulus of the matrix, $\varphi_{f}$ is the volume fraction of the fillers, and η is given by [46]:

(10) $$\eta =\frac{\left(E_{f}/E_{m}\right)-1}{\left(E_{f}/E_{m}\right)+\xi }\;\;\;,$$

where $E_{f}$ and $E_{m}$ are the elastic modulus of the filler and the matrix, respectively. The parameter ξ depends on the geometry of the fillers. For the transverse modulus, it has been reported that ξ=2 gives acceptable results. For the longitudinal modulus, ξ can be determined using the following Equation where D and L are the diameter and the length of the filler, respectively [46]:

(11) $$\xi =2\times \frac{L}{D}.$$

Recently, a new form of the Halpin-Tsai model was proposed for the prediction of the elastic modulus of CNT-reinforced polymer nanocomposites. An orientation factor, $f_{R}$, was incorporated into Equation (10) to account for the random dispersion of the CNT into the nanocomposites. A waviness efficiency factor, $f_{w}$, was added to account for the CNTs waviness. In addition, an agglomeration efficiency factor, $f_{A}$, was added to account for the CNTs agglomerated state [47]:

(12) $$\eta =\frac{\left(f_{R}f_{w}f_{A}E_{f}/E_{m}\right)-1}{\left(f_{R}f_{w}f_{A}E_{f}/E_{m}\right)+\xi }\;\;\;,$$

where $f_{w}$ and $f_{A}$ can be defined as follows [47]:

(13) $$f_{w}=1-\frac{A}{W},$$

(14) $$f_{A}=\exp\left(-\alpha \varphi_{f}^{\beta }\right).$$

Hassanzadeh-Aghdam et al. [47] have defined A and W as the amplitude and half-wavelength of a wavy CNT, respectively. The α and β parameters are related to the degree of agglomeration.

Hassanzadeh-Aghdam et al. [47] have illustrated that a more realistic prediction of the elastic modulus of CNT-PNCs was achieved using the new form of Halpin-Tsai model, which accounts for three important factors: CNT agglomeration state, waviness, and orientation.

### 3.2. Richeton-Ji Model for the Elastic Modulus

Wang et al. [21] used the Richeton model accounting for the effect of nanofillers. For this aim, the Richeton model was incorporated into the Ji model, also using the Takayanagi homogenization scheme [48]. The resulting three-phase model of Wang et al. [21], called the RJ model, relies on Ji et al. [49], who developed a model accounting for the interface bonding between the polymer matrix and the nanofillers. Therefore, the expressions of the RJ model for the reference instantaneous moduli of the composite $E_{ic}^{ref}$ as a function of the one of the polymer matrix $E_{im}^{ref}$ is given by [21]:

(15) $$\frac{E_{ic}^{ref}}{E_{im}^{ref}}={\left[\left(1-\alpha \right)+\frac{\alpha -\beta }{\left(1-\alpha \right)+\alpha \left(h-1\right)/\ln\left(h\right)}+\frac{\beta }{\left(1-\alpha \right)+\left(\alpha -\beta \right)\left(h+1\right)/2+\left(E_{f}/E_{im}^{ref}\right)}\right]}^{-1}.$$

Here, the three (i=1, 2, 3) $E_{ic}^{ref}$ are determined using Equation (4), and the three $E_{im}^{ref}$ are the instantaneous stiffness of the pure polymer at a reference frequency or strain rate. h is the stiffness ratio; $E_{f}$ is the Young’s modulus of the nanofillers; the $E_{ic}^{ref}$ can be calculated using Equation (15), and finally used in the Richeton model (Equation (2) or Equation (3)) to determine the modulus of the nanocomposites [21]. α and β are expressed as a function of the nanofillers volume fraction $\varphi_{f}$ [21]:

(16) $$\alpha =\sqrt{\left[2\left(\tau /t_{c}\right)+1\right]\varphi_{f}},\;\;\beta =\sqrt{\varphi_{f}},$$

where τ and $t_{c}$ are thicknesses for the interphase and particles, respectively.

**Figure 8:**
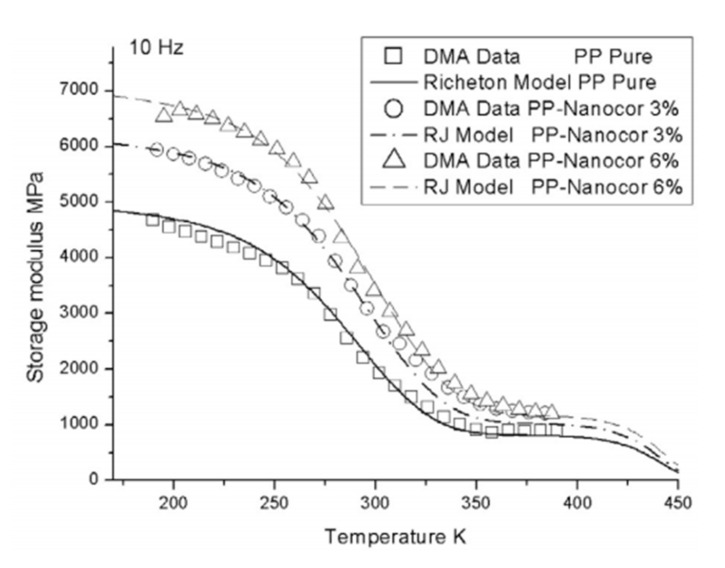
Prediction of storage modulus with RJ model compared to DMA measurements. Reprinted with permission from [21].

Wang et al. [21] have used the RJ model to predict the storage modulus and Young’s modulus as a function of temperature for polypropylene (PP) organoclay nanocomposites and under different frequencies/strain rates. The RJ model was validated by comparing results to experimental work done on PP/organoclay. Both the DMA and uniaxial compression test showed good agreement with the results obtained through RJ model for the storage modulus and Young’s modulus. As an example, Figure 8 shows the validation of the RJ model with DMA data for the storage modulus of PP (pure, 3 wt.%, and 6 wt.% nanoclay) at a frequency of 10 Hz.

In a recent paper by Acar et al. [37], they have modeled the elastic-plastic response of polymer-graphene nanocomposites. They have extended the Richeton model to include the effect of agglomeration of graphene nanocomposites by defining the three effective transition moduli ($E_{i}^{ref}$), which were taken as constant values in the work done by Richeton et al. [35].

### 3.3. The Richeton-Tandon-Weng Model for the Elastic Modulus

Tandon and Weng [50] have proposed a two-phase model (TW model) which predicts the elastic modulus of nanocomposites. Their work is based on the model developed by Mori and Tanaka [51]. Mori-Tanka (MT) model takes into consideration the effect of the nanofillers shape by accounting for the aspect ratio. In fact, Mori and Tanaka [51] have extended the inclusion model by Eshelby [52], where the aspect ratio was considered in the Eshelby tensor.

The Richeton-Tandon-Weng (RTW) model is the incorporation of Richeton model with TW model. The RTW model can be expressed as follows:

(17) $$\frac{E_{ic}^{ref}}{E_{im}^{ref}}=\frac{1}{1+\varphi_{f}\left[-2v_{m}A_{i3}+\left(1-v_{m}\right)A_{i4}+\left(1+v_{m}\right)A_{i5}A_{i}\right]/2A_{i}}.$$

Here, the parameters: $A_{i3}$, $A_{i4}$, $A_{i5}$, and $A_{i}$ are associated with the properties of the fillers and matrix and with the components of the Eshelby tensor. These parameters are determined from the $E_{im}^{ref}$ of the pure polymer. In addition, the $v_{m}$ is the Poisson ratio of the matrix. From Equation (17), the $E_{ic}^{ref}$, are determined and then, the $E_{ic}$ are calculated using Equation (4). Lastly, the modulus of the nanocomposite can be determined using the Richeton model.

Similar to the RJ model, Wang et al. [21] have predicted the storage modulus and Young’s modulus as a function of temperature for PP organoclay nanocomposites using the RTW model at different values of frequency/strain rate. The results of RTW model were found to be in reasonable agreement with the experimental results obtained from DMA (for storage modulus) and uniaxial compression test (for Young’s modulus).

Most of the studies take constant Poisson ratio; however, in the recent study by Bernard et al. [53], they have proposed to account for the effect of temperature and strain rates on the Poisson’s ratio through the following Equation:

(18) $$v\left(T,\dot{\varepsilon }\right)\;=\left\{\begin{array}{c} v_{0}+\left(v_{c}-v_{0}\right)\exp\left(-\frac{T-{\left(T_{g}\left(\dot{\varepsilon }\right)+\Delta T\right)}^{2}}{2\omega^{2}}\right)\;if\;T<T_{g}+\Delta T\\ v_{c}\;if\;T\geq T_{g}+\Delta T \end{array}\right..$$

Here, $v_{0}$ and $v_{c}$ are the Poisson’s ratio at the beginning and at the end of the glass transition region, respectively. The ΔT is half of the glass transition temperature region, and ω is a material parameter [53].

Table 1 presents a summary of the models used to predict the elastic modulus of polymers and polymer nanocomposites.

## 4. Yield Stress of Polymers and Polymer Nanocomposites

In this section, the models to predict the yield stress of polymers, as well as polymer nanocomposites, are discussed.

### 4.1. Yield Stress of Polymers

To predict the yield behavior of a polymer, Eyring [54] and Argon [55] proposed ones of the most well-known theories. The Argon and Eyring models were compared by Richeton et al. [56] with regard to experimental work done on PMMA and PC, and they reported that both are reasonably good, except only for restricted strain rate and temperature ranges. Consequently, there was a need for a further model expressing the yield stress within wide ranges of temperature and strain rate values: the cooperative model.

#### 4.1.1. Eyring Model

The yield stress depends significantly on temperature and strain rate [22]. Eyring [54] introduced one of the oldest theories, which is “a transition state theory”. This theory suggests that there is an activation energy barrier that must be overcome by the segments of the polymer chain at the yielding point. The yield stress, according to the Eyring theory, is expressed as [57]:

(19) $$\frac{\sigma_{y}}{T}=\frac{k}{V}\sin h^{-1}(\frac{\dot{\varepsilon }}{{\dot{\varepsilon }}_{0}\exp(-\frac{\Delta H}{RT})}).$$

Here, $\sigma_{y}$ is the yield stress, T is the temperature, R is the universal gas constant, V is the activation volume, ${\dot{\varepsilon }}_{0}$ is the pre-exponential constant factor, $\dot{\varepsilon }$ is the strain rate, and ΔH is the activation energy.

#### 4.1.2. Argon Model

Argon [55] proposed a theory that accounts for the intermolecular energy barrier to shear yielding, the yield stress ($\sigma_{y}$) is given by [56]:

(20) $$\sigma_{y}=\frac{0.077\;\sqrt{3}}{2}\frac{E}{1-\nu^{2}}{\left(1-\frac{kT}{\frac{0.77\;\sqrt{3}}{2}\frac{E}{1-\nu^{2}}\;V}\ln\left(\frac{{\dot{\varepsilon }}_{0}}{\dot{\varepsilon }}\right)\right)}^{6/5},$$

where ν is the Poisson’s ratio, E is the Young’s modulus, T is the temperature, k is the Boltzmann constant, V is the activation volume, ${\dot{\varepsilon }}_{0}$ is the pre-exponential constant factor, and $\dot{\varepsilon }$ is the strain rate. It is important to note that in this model, the Young’s modulus (E) is taken as constant for a given test temperature (T). A thermodynamic analysis was conducted by Richeton et al. [58] for the yield stress of amorphous polymers and showed that both Argon and Eyring models present nearly the same physical limitations with regard to the temperature and strain-rate response of yield stress.

#### 4.1.3. The Modified Argon Model

In a recent research work, Bernard et al. [59] proposed a new modification of the Argon model to extend its application to a broader range of temperature and strain rates. In their work, instead of using the constant Young’ modulus, as shown in Equation (14), they used the expression given by the Richeton model, Equation (3) where the Young’ modulus varies by temperature and strain rate:

(21) $$\sigma_{y}=\frac{0.077\;\sqrt{3}}{2}\frac{E\left(T,\dot{\varepsilon }\right)}{1-\nu^{2}}{\left(1-\frac{kT}{\frac{0.077\;\sqrt{3}}{2}\;\frac{E\left(T,\dot{\varepsilon }\right)}{1-\nu^{2}}\;V}\ln\left(\frac{{\dot{\varepsilon }}_{0}}{\dot{\varepsilon }}\right)\right)}^{6/5}.$$

Bernard et al. [59] showed that the modified Argon model could correctly predict the yield stress over a wider range of temperature and strain rate.

#### 4.1.4. The Ree-Eyring Model

The work by Bauwens [60], Bauwens et al. [61], Bauwens-Crowet [62], and Bauwens-Crowet et al. [63,64] have illustrated that in order to model the yield stress, two rheological phenomena need to be considered. The Ree-Eyring theory of non-Newtonian viscosity includes two activation processes, which are α and β [65,66]. The Ree-Eyring Equation expresses the yield stress under uniaxial loading, $\sigma_{y}$, as follows [66]:

(22) $$\frac{\sigma_{y}}{T}=A_{\alpha }\left(\ln\left(2C_{\alpha }\dot{\varepsilon }\right)+\frac{Q_{\alpha }}{kT}\right)+A_{\beta }\sin h^{-1}\left(C_{\beta }\dot{\varepsilon }\cdot \exp\left(\frac{Q_{\beta }}{kT}\right)\right).$$

Here, the activation parameters, $A_{i}$ and $C_{i}$ are for the two processes α and β. Similarly, the activation energies $Q_{i}$ are also for the two processes α and β. The other terms are as defined previously. The yield stress is dependent on both temperature and strain rate. Besides, Duckett et al. [67] and Truss et al. [68] have illustrated the dependency of yield stress on hydrostatic pressure.

Richeton et al. [56] have compared the three molecular-based theories: the state transition theory of the Ree-Eyring [54], the conformational change theory by Robertson [69], and the disclinations theory of Argon [55] for the prediction of yield stress for amorphous polymers. It was shown that these three models work for restricted ranges of temperature strain rates and that the Ree-Eyring model gives better prediction at higher strain rate values compared to the two other models.

#### 4.1.5. Cooperative Model: Governing Equations

A later paper by Richeton et al. [66] focused on the cooperative model. The cooperative model was first introduced by Fotheringham and Cherry [70,71]. They modified the original Eyring theory by first accounting for the internal stress as follows [66]:

(23) $$\sigma^{*}=\sigma_{y}-\sigma_{i},$$

where $\sigma^{*}$ is the effective stress, and $\sigma_{i}$ is the internal stress. The concept behind internal stress is that it accounts for defects resulting from previous thermal history. The internal stress adds up to the stress applied. Hence, it leads to faster yielding. The second modification is that yielding occurs when there is a cooperative movement of several polymer chain segments. Thus, the strain rate is given by [66]:

(24) $$\dot{\varepsilon }={\dot{\varepsilon }}^{*}\sin h^{n_{C}}\left(\frac{\sigma^{*}V}{2kT}\right),$$

where ${\dot{\varepsilon }}^{*}$ is the “characteristic strain rate”, and $n_{C}$ is a material constant which describes the motion of chain segments, and the other terms are as defined previously [66]. Therefore, the yield stress is as follows:

(25) $$\frac{\sigma_{y}}{T}=\frac{\sigma_{i}}{T}+\frac{2k}{V}\sin h^{-1}{\left(\frac{\dot{\varepsilon }}{{\dot{\varepsilon }}^{*}}\right)}^{1/n_{C}}.$$

Povolo and Hermida [72] and Povolo et al. [73] have shown that the cooperative model is more successful in the prediction of the yield stress of amorphous polymers, PMMA and PC, respectively, than the Ree-Eyring model under a wide range of temperature and strain rate. The same result was concluded by Brooks et al. [74] for the semi-crystalline polymer PE.

**Figure 9:**
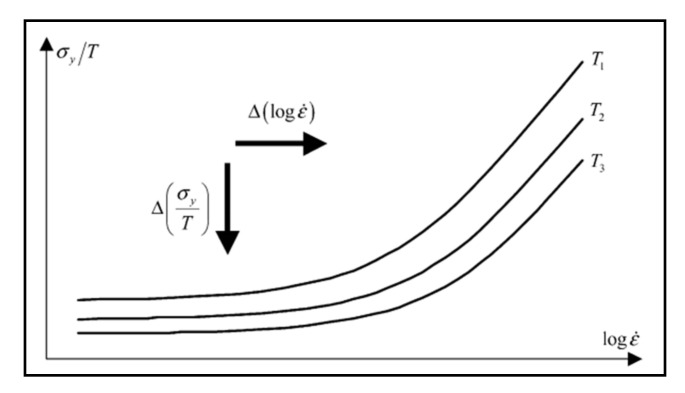
Eyring curves. Reprinted with permission from. Reprinted with permission from [66].

Richeton et al. [66] have used the superposition principle to determine $\sigma_{i}$ and ${\dot{\varepsilon }}^{*}$. As they have described, decreasing the temperature affects the yield stress in a similar way as increasing the strain rate and vice versa. As a result, the well-known time-temperature superposition principle can be used to establish a master curve for the Eyring plot. The Eyring plot shows curves for the reduced yield stress as a function of log $\dot{\varepsilon }$ and at different temperatures, as illustrated in Figure 9 [66].

As shown Figure 9, there are horizontal $\Delta \left(\log\dot{\varepsilon }\right)$ and vertical shifts $\Delta \left(\sigma_{y}/T\right)$ to establish a master curve at a reference temperature. The Equations for the shifts are as follows [37]:

(26) $$\Delta \left(\log\dot{\varepsilon }\right)\;=\log\dot{\varepsilon }\left(T_{ref}\right)-\log\dot{\varepsilon }\;\left(T\right)$$

(27) $$\Delta \left(\frac{\sigma_{y}}{T}\right)\;=\frac{\sigma_{y}\left(T_{ref}\right)}{T_{ref}}-\frac{\sigma_{y}\left(T\right)}{T}$$

Richeton et al. [66] used these shifts along with the expression for the reduced yield stress given by Equation (19) to have a physical meaning for the internal stress and the characteristic strain rate. Bauwens-Crowet et al. [63] proposed the superposition principle for the yield stress and used a “linearized Arrhenius law” to obtain expressions for both shifts. Hence, Richeton et al. [66] have used these expressions along with Equations they have determined for $\sigma_{i}$ and ${\dot{\varepsilon }}^{*}$ to obtain the following Equations:

(28) $$\left\{\begin{array}{c} {\dot{\varepsilon }}^{*}\left(T\right)\;={\dot{\varepsilon }}_{0}\exp\left(-\frac{\Delta H_{\beta }}{RT}\right)\\ \sigma_{i}\left(T\right)\;=\sigma_{i}\left(0\right)-m\cdot T \end{array}\right.\;,$$

where ${\dot{\varepsilon }}_{0}$ is “a constant pre-exponential strain rate”, m is a constant related to the material, $\sigma_{i}\left(0\right)$ is the internal stress at zero K, and $\Delta H_{\beta }$ is the beta activation energy (secondary relaxation). Richeton et al. [66] combined Equation (25) with Equation (28) to obtain the cooperative model for the yield stress for $T<T_{g}$, which is expressed as follows:

(29) $$\sigma_{y}=\sigma_{i}\left(0\right)-m\cdot T+\frac{2kT}{V}\sin h^{-1}{\left(\frac{\dot{\varepsilon }}{{\dot{\varepsilon }}_{0}\exp\left(-\frac{\Delta H_{\beta }}{RT}\right)}\right)}^{1/n_{C}}.$$

This model was validated by comparing the results with experimental work done on three amorphous polymers (PMMA, PC, and PVC). It was proven that the model is valid even at high strain rates. In fact, Richeton et al. [66] have extended the cooperative model to above $T_{g}$ and validated the model at high temperatures. Additionally, another paper by Richeton et al. [36] illustrated the influence of strain rate and temperature on the mechanical behavior of amorphous polymers. They did uniaxial compression tests (quasi-statics and dynamics) on three amorphous polymers (PMMA, PC, and polyamidimide (PAI)) and showed that elastic modulus, yield stress, and strain hardening rate all decrease with increasing temperature, and they all increase with increasing strain rate. Moreover, it is vital to mention that adiabatic heating affects strain hardening, especially at high values of strain rate. Strain hardening decreases substantially at a high strain rate because of the effect of adiabatic heating [36].

#### 4.1.6. Cooperative Model: Yield Stress for Semi-Crystalline Polymers

The cooperative model was used to predict the yield behavior of semi-crystalline polymers by Gueguen et al. [57]. This latter class of polymers consists of two phases: amorphous and crystalline. Therefore, to extend the cooperative model from Equation (23) for semi-crystalline polymers, the activation volume and activation energy will be the effective activation volume and effective activation energy. To this aim, Gueguen et al. [57] used the phenomenological law that was proposed by Takayanagi [75] as the homogenization method, as follows [22]:

(30) $$\left\{\begin{array}{c} \Delta H_{M}=\frac{\varphi \cdot \Delta H_{c}\cdot \Delta H_{a}}{\Omega \cdot \Delta H_{a}+\left(1-\Omega \right)\Delta H_{c}}+\left(1-\varphi \right)\Delta H_{a}\\ V_{M}=\frac{\varphi \cdot V_{c}\cdot V_{a}}{\Omega \cdot V_{a}+\left(1-\Omega \right)V_{c}}+\left(1-\varphi \right)V_{a} \end{array}\right..$$

Here, $\Delta H_{M}$ is the effective activation energy and $V_{M}$ is the effective activation volume for semi-crystalline polymers. $\Delta H_{c}$ and $\Delta H_{a}$ are the activation energy, respectively, of the crystalline and amorphous phases. $V_{c}$ and $V_{a}$ are the activation volume of the crystalline and amorphous phases. The parameters Ω and φ are related to the amorphous ($f_{a}$) and crystalline ($f_{c}$) volume [57]:

(31) $$\left\{\begin{array}{l} f_{c}=\varphi \cdot \Omega \\ f_{a}=1-\varphi \cdot \Omega \end{array}\right..$$

In the case of semi-crystalline polymers, Gueguen et al. [57] have reported a good agreement between the theoretical and experimental results for the cooperative model on polyethylene (PE) and polyethylene terephthalate (PET).

### 4.2. Yield Stress of Polymer Nanocomposites (Extended Cooperative Mode)

In the work done by Matadi Boumbimba et al. [22], an extension of the cooperative model was proposed for the yield stress of the following polymer nanocomposite: PP filled with organoclay. As the PP matrix is a semi-crystalline polymer matrix, the effective activation energy and effective activation volume for semi-crystalline PP are determined using the expression given in Equation (30). Similarly, in the reported composite approach, since the nanofillers contribute to the plastic deformation, Matadi et al. [76] have extended the expression of the effective parameters ($\Delta H_{eff}$ & $V_{eff}$) of the cooperative model after Takayanagi model [77]:

(32) $$\left\{\begin{array}{c} \Delta H_{eff}=\frac{\varphi \cdot \Delta H_{2}\cdot \Delta H_{1}}{\Omega \cdot \Delta H_{1}+\left(1-\Omega \right)\Delta H_{2}}+\left(1-\varphi \right)\Delta H_{1}\\ V_{eff}=\frac{\varphi \cdot V_{2}\cdot V_{1}}{\Omega \cdot V_{1}+\left(1-\Omega \right)V_{2}}+\left(1-\varphi \right)V_{1} \end{array}\right.\;\;.$$

Here, $\Delta H_{eff}$ is the effective activation energy and $V_{eff}$ is the effective activation volume for semi-crystalline polymers. $\Delta H_{1}$ and $\Delta H_{2}$ are, respectively, the activation energy of the matrix and fillers phases. $V_{1}$ and $V_{2}$ are the activation volume of the matrix and fillers phases, respectively. *Ω* and *φ* are parameters related to the volume fraction of the fillers.

#### 4.2.1. GMC Model

Matadi Boumbimba et al. [22] have presented two modified cooperative models; called the GMC and CBP models. The GMC model assumes that the polymer nanocomposite is a material of two phases: the matrix and the filler. In this model, the yield stress of nanocomposites is predicted as follows [76]:

(33) $$\frac{\sigma_{y,c}}{T}=\frac{\sigma_{i}\left(0\right)-m\cdot T}{T}+\frac{2k}{V_{eff}}\sin h^{-1}{\left(\frac{\dot{\varepsilon }}{{\dot{\varepsilon }}_{0}\exp\left(-\frac{\Delta H_{eff}}{RT}\right)}\right)}^{1/n_{C}}.$$

Here, $\sigma_{y,c}$ is the yield stress of the nanocomposite material, $\Delta H_{eff}$ and $V_{eff}$ are the effective activation energy and effective activation volume of the polymer nanocomposite, and the other parameters are defined after Equation (29).

The effective activation energy increases when the volume fraction of organoclay increases, while the effective activation volume decreases when the volume fraction of organoclay increases [22,76].

#### 4.2.2. CBP Model

Turcsányi et al. [78] have developed a relation to predict the yield stress of nanocomposite materials by considering the composite material to have a third phase that accounts for the extent of interfacial interaction as follows:

(34) $$\frac{\sigma_{y,c}}{\sigma_{y,M}}=\left[\frac{1-\varphi_{f}}{1+2.5\varphi_{f}}\;\exp\left(B\varphi_{f}\right)\right].$$

Here, $\varphi_{f}$ is the volume fraction of the fillers, B is a parameter that quantitatively measures the interphase/interface strength (B=0 for a weak matrix-fillers interaction), and $\sigma_{y,c},\sigma_{y,M}$ are the yield stress of the nanocomposite material and polymeric matrix, respectively.

Therefore, the CBP model considers the polymer nanocomposite to be a three-phase material where the third phase is the interphase between the matrix and the filler. The CBP model considers the nanofillers to be rigid elastic but accounts for matrix/fillers interphase as the third phase. By introducing the expression of $\sigma_{y,M}$ to the Equation above, the CBP model can be written as [22]:

(35) $$\frac{\sigma_{y,c}}{T}=\left[\frac{1-\varphi_{f}}{1+2.5\varphi_{f}}\;\exp\left(B\varphi_{f}\right)\right]\times \left[\frac{\sigma_{i}\left(0\right)-m\cdot T}{T}+\frac{2k}{V_{eff}}\sin h^{-1}{\left(\frac{\dot{\varepsilon }}{{\dot{\varepsilon }}_{0}\exp\left(-\frac{\Delta H_{eff}}{RT}\right)}\right)}^{1/n_{C}}\right],$$

where all terms have been defined previously. Matadi Boumbimba et al. [22] have found that both GMC and CBP models give good predictions for the yield stress when compared to the experimental results for PP organoclay nanocomposite. However, they suggested that the CBP model is better since it considers the presence of interphase.

In a recent paper, Majzoobi et al. [79] have proposed a modification to the first term in Equation (35) through the introduction of a second and a third parameter of *B* ($B_{2}$ and $B_{3}$) as follows:

(36) $$\begin{array}{c} \frac{\sigma_{y,c}}{T}=\left[\frac{1-\varphi_{f}}{1+2.5\varphi_{f}}\exp\left(B_{1}\varphi_{f}+B_{2}\varphi_{f}{}^{B_{3}}\right)\right]\times \\ \left[\frac{\sigma_{i}\left(0\right)-m\cdot T}{T}+\frac{2k}{V_{eff}}\sin h^{-1}{\left(\frac{\dot{\varepsilon }}{{\dot{\varepsilon }}_{0}\exp\left(-\frac{\Delta H_{eff}}{RT}\right)}\right)}^{1/n_{C}}\right]. \end{array}$$

**Figure 10:**
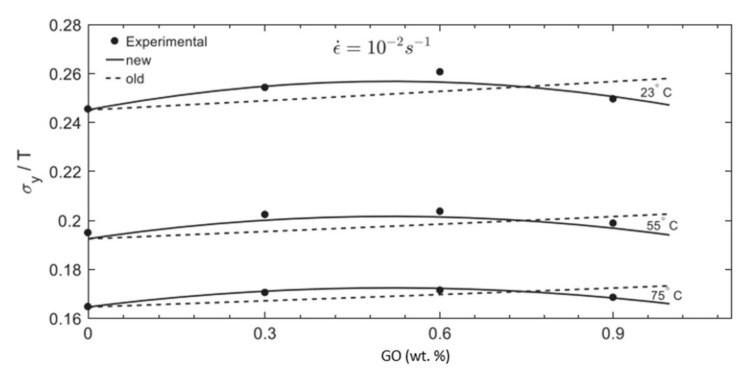
Yield stress/temperature ratio vs. GO weight fraction for $\dot{\varepsilon }=10^{-2}\;s^{-1}$. Reprinted with permission from [79].

According to Majzoobi et al. [79], this model adjustment helps improve the prediction of the agglomeration for nanofillers when the filler content increases. Figure 10 shows the prediction of the yield stress for polycarbonate reinforced by graphene oxide (GO) as a function of the weight fraction of GO for three different temperatures (23, 55, and 75 °C) at $\dot{\varepsilon }=10^{-2}\;s^{-1}.$

As illustrated in Figure 10, the prediction of the yield stress by the new model presented in Equation (36) fits better with the experimental data than the old model presented in Equation (29); especially for higher weight fraction of GO where the agglomeration of the nanofillers in the matrix increases.

Table 2 shows a summary of the models used to predict the yield stress for polymers and polymer nanocomposites.

## 5. Modeling the Porosity Effect on the Mechanical Behavior

Porosity is one of the critical parameters that influences the mechanical properties of different polymeric materials, such as polymeric composite foams used for bone tissue engineering and polymeric nanocomposites-based membranes used in water treatment applications [80,81,82]. In fact, different studies have focused on studying the effect of porosity, pore size, and pore size distribution on the mechanical properties of materials, especially ceramic and rocks [83,84,85]. Wagh et al. [84] have mentioned several semi-empirical formulas linking elastic modulus to the total porosity including:

(37) $$E\left(p\right)\;=E_{0}e^{\left(-bp\right)},$$

(38) $$E\left(p\right)\;=E_{0}\left(1+Ap/\left[1-\left(A+1\right)p\right]\right),$$

(39) $$E\left(p\right)\;=E_{0}\left(1-f_{1}p+f_{2}p^{2}\right),$$

where $E\left(p\right)$ is the Young’s modulus of a porous material, $E_{0}$ is the Young’s modulus of the nonporous material, and p is the porosity volume fraction. The constants $b,\;A,\;f_{1},\;\mathrm{and}\;f_{2}$ are all adjustable parameters. According to Wagh et al. [84], the problem with these formulas is that there is no correlation between the material’s microstructure and the material’s mechanical properties. In other words, there is no physical significance of the constants shown in these formulas.

A more interesting formulation linking porosity to the modulus of elasticity is the power-law model. The formulation, explanation, and significance of this model are given in the following sections.

### 5.1. Power Law for Modeling Porosity

Gibson and Ashby [86] have studied the three-dimensional mechanics of cellular solids (foams). They have discussed the mechanics of natural (e.g., wood) and human-made (e.g., foam polymers) cellular solids. Ashby [87] has discussed the typical stress-strain curve for a foam under compression. Ashby [87] has mentioned that there are three regions in the stress-strain curve, starting with the linear elastic region at low strain; then passing through a plateau of deformation region where the stress is almost constant, and the last region is called the densification region, which signifies a collapse of the cell walls completely. Gibson and Ashby [86] have analyzed how these three regions affect the behavior of three-dimensional cellular materials (both open-cell and closed-cell foam) and proposed models to determine the mechanical properties of foams.

The Gibson and Ashby model relates the linear elastic modulus with the relative densities as given by the following Equation [86]:

(40) $$\frac{E}{E_{s}}=C{\left(\frac{\rho }{\rho_{s}}\right)}^{n}.$$

Here, E and $E_{s}$ are the Young’s modulus of the foam (i.e., porous) material and the Young’s modulus of the solid (i.e., nonporous) cell wall material, respectively. Similarly, ρ and $\rho_{s}$ are the foam’s density and cell wall material’s density, respectively. Finally, *C* and *n* are both constants. Gibson and Ashby [86] have done experiments on three types of foams open-cell polyurethane (flexible), closed-cell foam polyurethane (rigid), and closed-cell foam polyethylene (flexible). They have shown that *C* = 1 through comparing experimental data with Equation (40). Ashby [87] has also shown that *C* = 1.

Blaker et al. [80] have used the model by Gibson and Ashby to relate elastic modulus to the porosity ($\varphi_{p}$) using the following expression:

(41) $$\varphi_{p}=1-\frac{\rho }{\rho_{s}},$$

(42) $$\frac{E}{E_{s}}=C{\left(1-\varphi_{p}\right)}^{n}$$

Hence, according to Bruno et al. [88], the parameter *n* in the power law is called the pore morphology factor. Wagh et al. [84] have reported that the parameter *n* depends on the tortuosity of the material, and Wong et al. [83] have stated that *n* is related to the pore size distribution of the material. According to Blaker et al. [80] and Roberts and Garboczi [85], the parameter *n* depends on the material’s microstructure. Both studies [80,85] have demonstrated that the general range value of *n* is from 1 to 4. Roberts and Garboczi [85] have stated that according to previous experimental results, the range for *n* is 1 < *n* < 2 in the case of a closed-cell porous structure, and *n* increases to 2 for open-cell porous structure. In addition, Gibson and Ashby [86] have illustrated that *n* = 2 in the case of open-cell porous isotropic structures. According to Ashby [87], most synthetic foams are practically isotropic and open-cell.

### 5.2. Modeling the Mechanical Behavior of Porous Polymers and Polymer Nanocomposites

A recently reported study has correlated the power-law model to the significant elastic modulus and yield stress models to predict the mechanical properties of porous polymeric-based materials and porous polymeric nanocomposites-based materials [89].

#### 5.2.1. Elastic Modulus

Alasfar et al. [89] have incorporated the effect of porosity into the model proposed by Richeton et al. [35] to investigate the elastic modulus of porous polymeric materials:

(43) $$\begin{array}{c} E\left(T,f,\varphi_{p}\right)\;=\{\left(E_{1}\left(f\right)\;-\;E_{2}\left(f\right)\right)\cdot \exp\left[-{\left(\frac{T}{T_{\beta }\left(f\right)}\right)}^{m_{1}}\right]\;+\;\left(E_{2}\left(f\right)\;-\;E_{3}\left(f\right)\right)\cdot \\ \exp\left[-{\left(\frac{T}{T_{g}\left(f\right)}\right)}^{m_{2}}\right]\;+\;E_{3}\left(f\right)\cdot \exp\left[-{\left(\frac{T}{T_{f}\left(f\right)}\right)}^{m_{3}}\right]\}\cdot {\left(1-\varphi_{p}\right)}^{n}. \end{array}$$

This model was applied to examine the storage modulus of porous PP. To investigate the effect of porosity on porous polymeric materials reinforced by nanofillers. The power-law model was also incorporated into the model proposed by Wang et al. [21] as follows:


(44) $$\left\{\begin{array}{c} \frac{E_{ic}^{ref}\left(\varphi_{p}\right)}{E_{im}^{ref}}={\left[\left(1-\alpha \right)\;+\;\frac{\alpha -\beta }{\left(1-\alpha \right)\;+\;\alpha \left(h-1\right)/\ln\left(h\right)}+\frac{\beta }{\left(1-\alpha \right)\;+\;\left(\alpha -\beta \right)\left(h+1\right)/2\;+\;\left(E_{f}/E_{im}\right)}\right]}^{-1}{\left(1-\varphi_{p}\right)}^{n}\\ E\left(T,f,\varphi_{p}\right)\;=\;\left(E_{1c}\left(f,\varphi_{p}\right)\;-\;E_{2c}\left(f,\varphi_{p}\right)\right)\cdot \exp\left[-{\left(\frac{T}{T_{\beta }\left(f\right)}\right)}^{m_{1}}\right]\\ +\left(E_{2c}\left(f,\varphi_{p}\right)\;-\;E_{3c}\left(f,\varphi_{p}\right)\right)\cdot \exp\left[-{\left(\frac{T}{T_{g}\left(f\right)}\right)}^{m_{2}}\right]\\ +E_{3c}\left(f,\varphi_{p}\right)\cdot \exp\left[-{\left(\frac{T}{T_{f}\left(f\right)}\right)}^{m_{3}}\right]. \end{array}\right.$$

All parameters in this model are as defined in the previous section. This model was applied to the porous PP reinforced by nanoclay. Both models have shown that the existence of pores has lowered the storage modulus for both pure PP and PP-organoclay nanocomposites.

**Figure 11:**
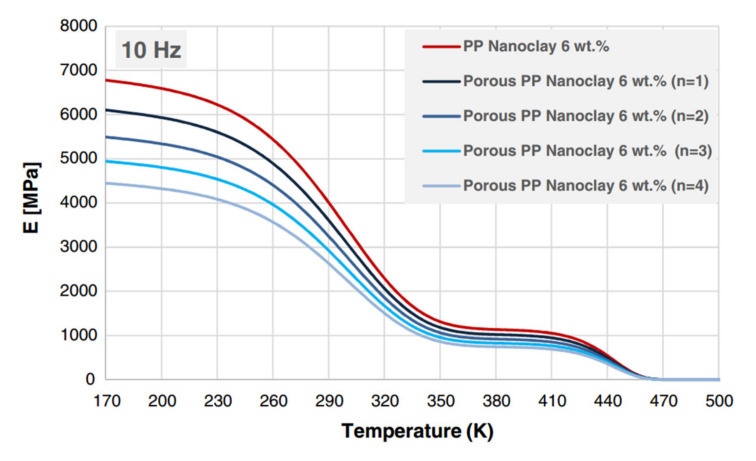
Sensitivity analysis on the pore morphology factor. Reprinted with permission from [89].

Regarding the pore morphology factor (n), Alasfar et al. [89] have conducted a sensitivity analysis to investigate the effect of the parameter (n) on the storage modulus of porous PP-organoclay nanocomposites. The (n) values chosen for the sensitivity analysis were 1, 2, 3, and 4 since the reported range for n is between 1 and 4. Figure 11 illustrates that as the value of the parameter (n) increases from 1 to 4, so does the stiffness of the porous PP with 6 wt.% nanoclay [89]. 

#### 5.2.2. Yield Stress

The modified cooperative model by Matadi Boumbimba et al. [22], CBP model, has been recently extended to account for the effect of porosity similarly using the power-law model [89]:

(45) $$\begin{array}{c} \frac{\sigma_{y,c}}{T}=\left[\frac{1-\varphi_{f}}{1+2.5\varphi_{f}}\exp\left(B\varphi_{f}\right)\right]\times \\ \left[\frac{\sigma_{i}\left(0\right)-m\cdot T}{T}+\frac{2k}{V_{eff}}\sin h^{-1}{\left[\frac{\dot{\varepsilon }}{{\dot{\varepsilon }}_{0}\exp\left(-\frac{\Delta H_{eff}}{RT}\right)}\right]}^{1/n_{C}}\right]\times {\left(1-\varphi_{p}\right)}^{n}. \end{array}$$

**Figure 12:**
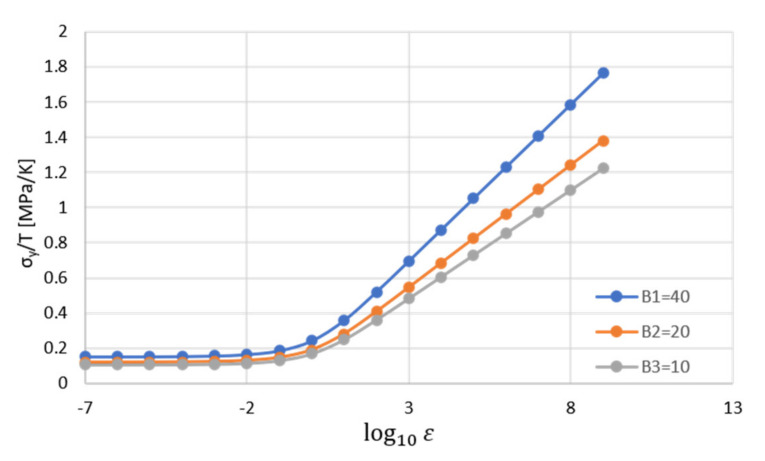
The effect of parameter B on the yield stress of PP 3 wt.% nanoclay. Reprinted with permission from [89].

In the case of a pure porous polymer, the first term is equal to one as $\varphi_{f}=0$ and B=0, as explained by Matadi Boumbimba et al. [22]. Figure 12 illustrated the effect of varying the parameter B on the yield stress of porous ($\varphi_{p}=5\%$) PP reinforced by 3 wt.% nanoclay [89].

**Figure 13:**
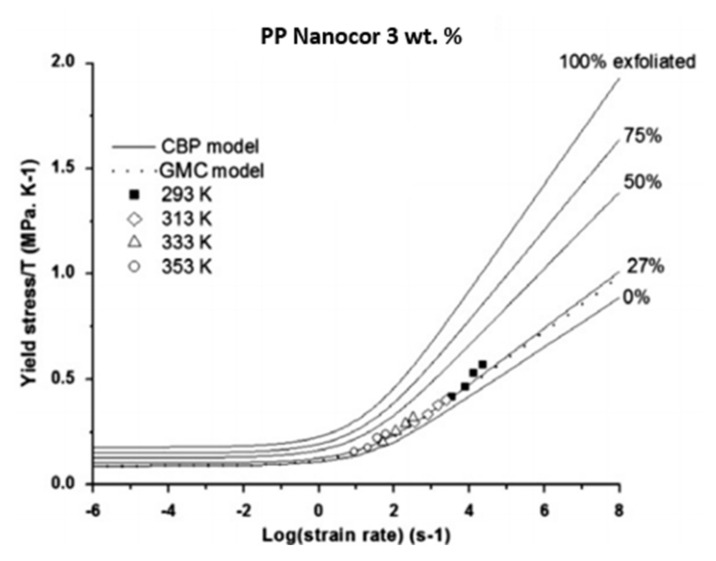
Yield stress/temperature versus log (strain rate) [22].

Figure 12 demonstrates that the larger the parameter B is, the higher the yield stress of porous PP nanoclay. According to Alasfar et al. [89], the explanation behind this behavior is the fact that a higher exfoliation degree of PP/nanoclay is represented by a larger value of parameter B. According to Matadi Boumbimba et al. [22], determining the parameter B can be useful in estimating the extent of exfoliation. They illustrated that a 3 wt.% organoclay concentration corresponds to an exfoliation degree of 27%, as shown in Figure 13.

## 6. Three Dimensional Computational Implementation to the Elastic-Plastic Stress-Strain Curve

The original work of Boyce et al. [90] has demonstrated the implementation of the Argon model into a three-dimensional constitutive model. This model describes the elastic-viscoplastic deformation for glassy polymers. It incorporates the yield and post-yield behavior of glassy polymers and takes into account the effect of strain rate, temperature, pressure, strain softening, and strain hardening. Arruda and Boyce [91] have shown that this constitutive model is capable of predicting the stress-strain behavior of the two amorphous polymers, PC and PMMA, from low to moderate strain rates. Boyce et al. [92] have also demonstrated the successful ability of the constitutive model to predict the behavior of PC polymer under different states of deformation (simple shear, uniaxial compression and plane strain compression, and uniaxial tension). They compared simulated prediction to the experimental results. Wu et al. [93] used this modeling approach to predict the stress-strain response, under large compressive deformation, of a non-filled and silica-filled resin.

Later on, Mulliken and Boyce [94] extended the model to predict amorphous polymers’ behavior for high strain rates and a wide range of temperature, using the Ree-Eyring concept for the yield behavior. The 3D constitutive model was numerically implemented into ABAQUS through a user material subroutine. It was shown that the model has successfully predicted the linear elasticity, yield behavior, strain softening, and strain hardening for the polymer PC. However, the problem with the Mulliken and Boyce model is that it is not valid through the glass transition temperature. Besides, the effect of adiabatic heating needs to be incorporated since it is an essential aspect of material deformation behavior. Therefore, Richton et al. [58] have proposed the use of the cooperative model for the yield stress in the 3D modeling. This formulation is valid from a glassy to rubbery region under a wide range of temperature and strain rate, and it took into consideration the effect of adiabatic heating.

To account for the strain softening observed on the stress-strain curves after the onset of yielding, a phenomenological relation was proposed by Boyce et al. [90]. The softening evolution of the shear resistance, $\dot{S}$, can be expressed as follows [93]:

(46) $$\dot{S}=h_{s}\left(1-\frac{S}{S_{ss}}\right){\dot{\varepsilon }}^{p}\;.$$

Here, $h_{s}$ is the softening slope, S is the material current state of athermal deformation resistance, and $S_{ss}$ is the steady state value of S [58,90,93]. In this case, the Argon’s model is expressed as follows:

(47) $$\sigma_{y}=S{\left[1-\frac{\left(1-v\right)T}{0.076\;GV}\ln\left(\frac{{\dot{\varepsilon }}_{0}}{{\dot{\varepsilon }}^{p}}\right)\right]}^{\frac{6}{5}}.$$

where the initial value of athermal resistance, $S_{0}$, can be defined as:

(48) $$S_{0}=\frac{0.076G}{\left(1-v\right)},$$

here, G is the shear modulus, and v is the Poisson ratio.

To account for this strain softening in the case of the cooperative model, Richeton et al. [58] proposed a similar phenomenological relation, but applied to the evolution of the internal stress in the cooperative model. In this work, Richeton et al. [58] have also added the effect of hydrostatic pressure in the evolution relation of the internal stress.

As was shown in Figure 3, the stress-strain response shows a strong strain hardening region at large strains when the polymer is deformed at a temperature below the glass transition. This hardening is due to molecular orientation and is similar to the hardening observed at large strains when the polymer is deformed above the glass transition (rubbery behavior). To model the hardening in glassy polymers, a flow rule with kinematic hardening was proposed by Parks et al. [95] and Boyce et al. [90]. In these works, they used a statistical rubber-elasticity model based on the tree-chain approach to compute the back stress due to molecular orientation. This statistical rubber elasticity modeling of the back stress for molecular orientation resistance was later extended to the eight-chains modeling approach for rubber-elasticity by Arruda and Boyce [96]. This eight-chain theory was then used by several authors to model the orientation hardening during large deformation of glassy polymers (see [58] and references therein). The formulation based on Richeton et al. [58] was implemented into the finite element code Abaqus by Bernard et al. [53]. In more recent work, Bernard et al. [97] proposed a generalized 3D formulation based on the strain-strain duality to correctly account for large deformation kinematics. To account for the hardening due to molecular orientation, another method proposed by Çolak et al. [98] was successfully used to predict the stress-strain response of PMMA. This approach, called the cooperative-VBO model, is based on implementing the cooperative model into the 3D elastic-viscoplastic formulation of the overstress model (see Çolak et al. [98] and references therein). This cooperative-VBO model was also extended, in a composite formulation, to predict the elastic-viscoplastic behavior of polymer nanocomposites by Acar et al. [37]. Other important works in this 3D computational work of large deformation behavior of polymers were proposed by Naït-Abdelaziz and co-workers for silica/polymer nanocomposites as well as for semi-crystalline polymers [99,100].

At relatively high strain rates, the temperature of the plastically deformed polymer samples increases due to adiabatic heating. Since polymers are very sensitive to temperature changes, the 3D constitutive model accounts for heat generated during plastic deformation of a polymer, which is known as adiabatic heating [101,102]. As previously mentioned, adiabatic heating affects strain hardening and strain softening, especially at high strain rate values [36]. The Equation for general energy balance can be written as [58]:

(49) $$\dot{q}=\rho \left(T\right)\;\cdot \;c_{p}\left(T\right)\;\cdot \;\dot{T}-\mathrm{div}\left(\Gamma \;\cdot \;\mathrm{grad}\left(T\right)\right),$$

where $\dot{q}$ is the rate of heat generated due to plastic flow, $\rho \left(T\right)$ is the density, $c_{p}\left(T\right)$ is the specific heat, and Γ is the thermal conductivity [91,103]. Unlike in metals, where it is well established that only 90% of the plastic work is converted to heat, this percentage is not clearly defined for polymers.

## 7. Experimental Characterization of Mechanical Behavior of Polymers and Polymer Nanocomposites

The aim of this section is to briefly discuss the main experimental characterization techniques used to investigate the mechanical behavior of polymeric materials and nanocomposites. It is important to note that a detailed review of the experimental characterization of the mechanical behavior is beyond the scope of this review paper. The purpose here is to highlight the important experimental tools necessary to validate models’ prediction and obtain critical material parameters needed for the modeling approach. 

As reported by Wang et al. [104] and Wang et al. [105], particular care has to be taken using conventional tests as these are difficult to directly apply to study the mechanical properties of membranes for water treatment because of their small thickness. However, in this section, the widely used mechanical tests are briefly addressed without going into details of the thin versus thick samples.

Dynamic mechanical analysis (DMA) is a powerful method that applies cyclic stress or cyclic deformation, and the corresponding strain or stress, respectively, is measured. The DMA test is a well-known tool to measure the mechanical properties as a function of frequency, temperature, and time [106]. In DMA, a sinusoidal deformation is applied to the material sample, and the response is measured. The thermal transitions, including secondary transition and glass transition temperature of polymers, can be detected using the DMA approach [107]. Hence, both storage modulus and loss modulus can be measured as a function of temperature at different frequencies. Ghasemi et al. [108] have prepared glass-fiber-reinforced PP and incorporated graphene nanoparticles at different loading. DMA test was used to investigate the effect of graphene nanoparticles on the storage modulus and showed a significant improvement in the modulus with the addition of 1 wt.% of nanoparticles. The stress-strain curve can be obtained, which will provide information about the elastic behavior (Young’s modulus and yield stress) as well as the viscoelastic behavior of the polymeric material. Many researchers use this tool to validate the predicted results of the models reviewed in this paper, such as in the work by [21,35,66] and others.

Uniaxial testing is one of the most common methods used to determine Young’s modulus, yield stress, ultimate tensile stress, fracture toughness, and elongation at break [104]. All these properties can be determined via reporting the stress-strain curve. Moy et al. [30] have investigated the stress-strain response for PMMA under the uniaxial compression test. For quasi-static and intermediate strain rates, they used servo-hydraulic Instron test frame to perform the experiment. For the dynamic uniaxial compression test, a split-Hopkinson pressure bar (SHPB) was used for investigating the mechanical behavior of PMMA at high strain rates and under room temperature.

Similarly, Richeton et al. [36] have conducted uniaxial compression tests on three amorphous polymers (PAI, PC, and PMMA) under a wide range of strain rates and temperatures. In their work, SHPB was used to conduct the high strain rate experiments. The results of these tests provide information about the compressive yield stresses for a wide range of temperatures and strain rates, which is necessary for developing models that can accurately predict the mechanical behavior of the polymers. Uniaxial tensile testing is commonly used for porous polymeric membranes by uniaxially stretching the sample at a constant speed while both ends are gripped [104]. Both the load (force) and speed can be controlled. Reiter and Major [109] performed uniaxial tensile tests on PP from low to high strain rates and used the international standard for the tensile test to ensure reliable prediction for the FEM simulation and modeling.

Nanoindentation is a method that can be used to determine the modulus, fracture toughness, and hardness of material [104]. In the indentation test, the applied force into a specimen by an indenter and the corresponding displacement are measured. The stiffness of the material can then be determined from the load (force)-displacement curve. This method is considered effective for thin films and materials with small volumes [104]. Zhang et al. [110] have extracted the PMMA parameters in the model through fitting indentation test results. They showed that the FE-simulation results agree with the experimental results using these parameters [110,111]. Wang et al. [112] have used a nano-indenter to measure the modulus and hardness for a complex nanohybrid polymeric membrane and showed the effect of incorporation of graphene oxide on increasing both the modulus and hardness of the membrane.

## 8. Modeling of the Mechanical Behavior of Polymer Nanocomposites: Application Cases

The models reviewed in this paper for predicting the mechanical behavior of porous polymers and polymer nanocomposites can be used for different applications. In water treatment applications, porous polymer membranes have been widely employed due to their high efficiency and low cost [113]. Polyvinylidene fluoride (PVDF), polyethersulfone (PES), and polysulfone (PSF) have been most often used for the fabrication of micro-and ultrafiltration membranes owing to their mechanical, thermal, and chemical properties. In general, polymer membranes are required to have good mechanical strength, stability, and durability in addition to controllable porosity and filtration functionalities. The mechanical behavior of polymer nanocomposite membranes can be notably enhanced by incorporating a target nanofiller, and the nanofiller loading and distribution within the membrane matrix largely affect the total porosity and porous morphology of the prepared nanocomposite membranes. For example, Zhong and coworkers [114] prepared membranes from PSF blended with sulfonated polysulfone (SPSF) and reinforced by cellulose nanofillers (CNF). In another study, PSF was blended with surface modified CNF (M-CNF) after the addition of excess M-CNF [115]. Kamal et al. [116] prepared PSF/PVP membranes and incorporated halloysites nanotubes (HNTs) into the matrix. These studies showed that a specific addition of the nanofiller enhances the mechanical properties of the prepared membrane. In the paper by Zhang et al. [115], the addition of 0.4 wt.% M-CNF resulted in the highest tensile strength of the composite membrane. In the paper by Kamal et al. [116], the optimal HNTs loadings leading to the maximum elastic modulus and yield stress were 0.2 wt.% for the PSF matrix and 0.3 wt.% for the PSF/PVP matrix. However, Manawi et al. [117] have reinforced PSF with carbon nanotubes (CNT), and they showed that the mechanical properties (yield stress and Young’s modulus) dropped with the increase in the CNT content as a result of higher porosity in the fabricated composite membrane samples.

The importance of having good mechanical strength is dictated by the fact that the porous membrane might experience physical compaction when exposed to operating pressure, which severely deteriorates the membrane performance. In addition, polymeric membranes with low mechanical strength may fail at high operating pressure and during backwashing cleaning. Because the membranes are subjected to cycles of pressure and temperature changes during operation and cleaning; thus, it is necessary to investigate how the polymeric membranes behave under stress conditions similar to the ones in operation [104]. As a result, it is important to examine the membranes’ capability to endure deformation mechanisms to predict their lifetime. The mechanical properties essential for the membrane application in water treatment include the elastic modulus and yield stress. Hence, the discussed prediction models could be useful to foresee the mechanical properties of polymeric/polymeric nanocomposite-based membranes, which are essential for their efficient long-term performance. The experimental characterization techniques described in Section 7 can be effective for validating these models as well as determining the critical material parameters needed for the models.

In addition, modeling of the mechanical properties of nanocomposites could facilitate the development of novel scaffold materials such as polyglycolide and polylactide incorporated with hydroxy apatite (HA), bioactive glass or other inorganic particles in bone tissue engineering [80]. Poly-*d*-l-lactide (PDLLA)/nano-HA is a common porous composite that is considered as a biological scaffold material. This composite material exhibits shape memory property under uniaxial compression or bending test, which is an important property when this scaffold is implemented into the body [118]. A material with shape memory property can return to its original shape after temporarily being deformed by an external stimulus (e.g., light, temperature, or pH) [119]. Zheng et al. [120] have prepared PDLLA/HA composites and examined their mechanical properties in addition to their shape memory effect through DMA. Han et al. [118] have theoretically investigated the mechanical behavior of porous PDLLA/nano-HA through four hyper-elastic constitutive models and validated the models with results from the uniaxial compression experiment. According to Meskinfam [121], evaluating the mechanical properties is an essential step in the development of scaffolds. The mechanical properties that are important to evaluate include elastic and flexural modulus, tensile and compressive strength, and maximum allowable strain. It is vital to mention that the pore morphology and porosity largely influence the mechanical properties of scaffolds. Hence, accounting for the porosity and pore morphology effect in mechanical modeling is a must for the application of bone tissue engineering.

## 9. Conclusions

Polymers are widely used for various engineering applications, especially polymers reinforced by nanofillers, which increase their functionality and significantly improve their mechanical, thermal, and electrical properties. Hence, studying the mechanical properties of polymers and polymer nanocomposites is essential to decide on the most efficient polymeric/polymeric nanocomposite material needed for a specific application. Computational modeling is a useful tool to investigate the mechanical properties of polymers and polymer-based nanocomposites (both dense and porous). Reliable, accurate mechanical models can significantly reduce the number of experiments conducted and hence save time, effort, and cost.

This paper has presented a review of essential models widely used to predict the elastic behavior and yield stress of polymers and polymer reinforced by nanofillers. In terms of the elastic behavior, the Mahieux and Reifsnider model, the Richeton model, the Richeton-Ji model, and the Richeton-Tandon-Weng model have been discussed. For the yield stress, the Eyring model, Argon model, modified Argon model, Ree-Eyring model, and the cooperative model (both general and extended model for the cases of semi-crystalline polymers and polymer nanocomposites) have been reviewed. Results from different modeling studies on polymer nanocomposites have been reviewed as well.

This paper also reviewed the widely used models for predicting the porosity in terms of the elastic modulus. The recent models used to predict the elastic modulus and yield stress of porous polymers/polymer nanocomposites are discussed. The computational implementation of the elastic-plastic stress-strain curve has also been reviewed.

The review paper also briefly summarizes the experimental characterization techniques most used to investigate the mechanical behavior of polymeric/polymeric nanocomposite materials. The purpose is to highlight the important experimental tools necessary to validate the models’ prediction and obtain critical material parameters needed for the modeling approaches. The experimental characterization techniques reviewed in this paper are the DMA method, uniaxial test (compression and tension), and nanoindentation method.

Finally, the recent models that were reviewed in this paper for predicting the mechanical behavior of porous polymers and polymer nanocomposites can be used for different applications. In the field of water treatment, porous membranes made of polymeric/polymeric nanocomposite materials are usually used due to their wide commercial availability, low cost, and moderate efficiency. Porous polymer nanocomposites can also find their application in the field of bone tissue engineering as scaffold materials.

Based on this review paper, the authors would like to recommend the following for future work:

- The recent models used to predict the elastic modulus and yield stress of porous polymers/polymer nanocomposites have performed a sensitivity analysis on the pore morphology factor (*n*). More investigation on this parameter can be helpful to determine its value depending on the microstructural features of the chosen material;
- There is a need to conduct experiments on different porous polymeric materials or polymeric nanocomposite materials. This will help in increasing the validity of the models for the prediction of the elastic modulus and yield stress of porous polymers/polymer nanocomposites. Future experiments can include DMA tests as well as uniaxial compression/tension tests;
- The model given by Equation (45) for the yield stress prediction can be further modified by considering the three parameters of *B* presented in Equation (36). A study can be done to investigate how this modification can improve the prediction of the agglomeration for nanofillers.

## Figures and Tables

**Table 1:** A summary of the models * used for elastic modulus prediction.

Models	Equations	Parameters *	Use	Effect of $T,f/\dot{\varepsilon }$
Mahieux and Reifsnider model	Equation (1)	$m_{i},E_{i},T_{\beta },\;T_{g},T_{f}$	Polymers	Account for the effect of T
Richeton model	Equation (2) for storage modulus Equation (3) for Young’s modulus	$E_{i}\left(\dot{\varepsilon }/f\right)$ $T_{\beta }\left(\dot{\varepsilon }/f\right),$ $T_{g}\left(\dot{\varepsilon }/f\right),$ $T_{f}\left(\dot{\varepsilon }/f\right)$	Polymers	Account for the effects of $T,f/\dot{\varepsilon }$
Halpin-Tsai model	Equation (9)	$\xi ,E_{f},\varphi_{f}$	Polymer nanocomposites	Doesn’t account for the effects of $T,f/\dot{\varepsilon }$
RJ model	Equation (15)	$\varphi_{f},\tau /t_{c},h$ $E_{f}$	Polymer nanocomposites	Account for the effects of $T,f/\dot{\varepsilon }$
RTW model	Equation (17)	$\varphi_{f}v_{m},\;A_{i3}$ $,A_{i4}$ $,A_{i5}$ $,A_{i}$	Polymer nanocomposites	Account for the effects of $T,f/\dot{\varepsilon }$
Alasfar and co-workers’ model	Equation (43) Equation (44)	$\varphi_{p},\;n$ in addition to parameters of the Richeton and RJ models	Porous polymers Porous polymer nanocomposites	Account for the effects of $T,f/\dot{\varepsilon }$

* The material parameters in the models are usually measured experimentally, but some are parameters that are used as fitting parameters. The parameters in this table are defined within the paper.

**Table 2:** A summary of the models used for yield stress prediction.

Models	Equations	Parameters *	Use	Remarks
Eyring model	Equation (19)	${\dot{\varepsilon }}_{0},\;V,\;\Delta H$	Polymers	
Argon model	Equation (20)	${\dot{\varepsilon }}_{0},\;V,\nu \;$ $E\;\left(\mathrm{constant}\right)$	Polymers	
Modified Argon model	Equation (21)	${\dot{\varepsilon }}_{0},\;V,\;$ $E\;\left(T,\dot{\varepsilon }\right)$	Polymers	
Ree-Eyring model	Equation (22)	$A_{i},\;C_{i},Q_{i}$ i=α,β	Polymers	
Cooperative model	$T<T_{g}$: Equation (29)	$\sigma_{i}\left(0\right),m$ $,V,\;\Delta H_{\beta },\;{\dot{\varepsilon }}_{0},n_{c}$	Polymers	Compared to previous models, this model is the most accurate model for yield stress prediction
Extended cooperative model	GMC: Equation (33)	$\sigma_{i}\left(0\right),{\dot{\varepsilon }}_{0},m$ $,\Delta H_{eff},\;V_{eff},n_{c}$	Polymer nanocomposites	Two-phase
	CBP: Equation (35)	$\sigma_{i}\left(0\right),{\dot{\varepsilon }}_{0},m$ $,\Delta H_{eff},\;V_{eff},n_{c}$ $\varphi_{f},B$		Three-phase (the third phase is the interphase between matrix and fillers)
Alasfar and co-workers’ model	Equation (45)	$\varphi_{p},\;n$ in addition to parameters of the extended cooperative model (CBP)	Porous polymer nanocomposites	Three-phase (the third phase is the interphase between matrix and fillers)

* The material parameters in the models are usually measured experimentally, but some are used as fitting parameters. All parameters in this table are defined within the paper.

## Data Availability

Not Applicable.
